# Algorithm for the Simultaneous Measurement of Multiple Parameters Based on Wavelength Modulation Spectroscopy

**DOI:** 10.3390/s26051585

**Published:** 2026-03-03

**Authors:** Xiangyu Zhong, Qing Shi, Buqiang Zhang, Huiwen Niu, Gui Meng, Jianfa Zhou, Yongqing Peng

**Affiliations:** Beijing Research Institute of Telemetry, Beijing 100076, China; zxy12303006@163.com (X.Z.);

**Keywords:** confined space, TDLAS, multi-parameter, simultaneous measurement

## Abstract

To ensure personnel safety and prevent serious accidents, it is crucial to monitor parameters such as temperature, pressure, and gas composition concentrations in confined spaces. This study proposes a multi-parameter simultaneous inversion algorithm based on tunable diode laser absorption spectroscopy (TDLAS). The algorithm integrates the Levenberg–Marquardt (L-M) fitting method, single-line thermometry and manometry methods, spectral separation, and alternating iteration techniques, with an adaptive feedback mechanism adding to enhance convergence stability. Through this approach, simultaneous inversion of H_2_O, CO_2_, CO, and O_2_ concentrations, temperature, and pressure was successfully achieved. Simulation results demonstrated that the measurement accuracy meets practical requirements. This study provides an effective monitoring method for multi-parameter detection in confined spaces within conventional environments and lays a foundation for expanding the application scope of TDLAS technology.

## 1. Introduction

With rapid economic development and societal advancements, workplace environments have undergone significant transformations. An increasing number of individuals now engage in production and daily activities within confined spaces. Defined as enclosed areas with high isolation from the external environment, restricted entry or exit access, and inadequate ventilation [1], typical confined spaces include urban civil air defense facilities, pipeline networks, subway systems, industrial settings (e.g., flues, mining shafts, chemical reaction tanks), as well as specialized environments like submarine cabins, spacecraft modules, and space stations. Consequently, continuous monitoring of environmental parameters such as temperature, pressure, and gas composition concentrations within these spaces becomes imperative to ensure personnel safety and prevent catastrophic accidents.

Tunable Diode Laser Absorption Spectroscopy (TDLAS) utilizes a tunable diode laser whose output wavelength is modulated by adjusting the input current or temperature. This enables precise scanning of single or multiple gas molecular absorption lines, thereby obtaining high-resolution absorption spectra for extracting gas parameters through spectral analysis [2]. Compared with conventional methods, TDLAS exhibits superior sensitivity, rapid response [3], and immunity to cross-interference from coexisting gases, making it particularly suitable for simultaneous multi-component and multi-parameter measurements [4,5]. Goldenstein et al. [6] developed a compact fiber-optic probe integrating reflection and reception units, enabling synchronous temperature, pressure, and H_2_O concentration measurements in combustion flames. Parker et al. [7] designed a TDLAS gas sensor for monitoring gases in space launch systems. Mudgett et al. [8] first applied an experimentally developed TDLAS multi gas detection device on US Navy submarines. Based on this research, the following year they developed the second-generation TDLAS multi gas detector for the US Gastrointestinal Clinical Trial Program, which is used for gas component measurement in the medical field [9]. Oliver et al. [10] proposed a multi-species molar fraction and temperature sensor for in situ exhaust gas diagnosis of internal combustion engines. Based on TDLAS technology, temperature and six gas components (H_2_O, CO_2_, CO, CH_4_, NO_2_, and NO) were simultaneously measured through dual line temperature measurement. Séan et al. [11] developed a single-ended laser absorption sensor for the diagnosis of rotary detonation engines (RDEs), achieving in-situ time-resolved measurements of temperature, H_2_O, CO_2_, and CO concentrations. Mathews et al. [12] conducted high bandwidth measurements on (1) temperature, pressure, and CO, as well as (2) temperature and H_2_O in the annular combustion chamber of a rotating detonation rocket engine (RDRE). Shen et al. [13] realized continuous monitoring of toxic gases in mining environments with detection limits below 10^−4^, stability of 0.137%, and less than 5% deviation from third-party validation results.

Based on the analysis of the current research status described above, current multi-parameter measurement methods based on TDLAS include the following: (1) Multi-parameter detection of a single gas species, which measures parameters such as temperature, pressure, and concentration of a single gas along the laser path, as demonstrated in the study by Zhou et al. [14]. (2) Detection of temperature and concentration of multiple gas components, which determines the concentration and temperature of gas mixtures through absorption spectroscopy but requires additional sensors for pressure measurement to provide other environmental parameters for the inversion of temperature and component concentrations (e.g., the study by Meshcherinov et al. [15]). This requires that the response frequency of the pressure sensor measurements corresponds to the inversion speed of temperature and component concentrations. However, achieving a perfect correspondence between the two is difficult, and a time discrepancy is always present. Therefore, these methods have limitations, and none have truly achieved the simultaneous measurement of environmental parameters such as temperature and pressure along with gas component concentrations. To overcome these limitations, this study proposes a multi-parameter adaptive inversion algorithm based on TDLAS. This algorithm can directly and simultaneously invert temperature, pressure, and multiple gas concentrations from spectral information without the need for auxiliary sensors, thereby meeting the measurement requirements of practical applications.

TDLAS can be categorized into two primary techniques: Direct Absorption Spectroscopy (DAS) and Wavelength Modulation Spectroscopy (WMS). While DAS offers a straightforward working principle, its sensitivity limitations render it unsuitable for low-concentration gas detection. In contrast, WMS employs a high-frequency modulation signal superimposed on a low-frequency scanning waveform, which effectively suppresses low-frequency 1/*f* noise and other interference from detection devices, thereby reducing the detection limit of the gas concentration [16]. Given that direct absorption methods are highly susceptible to noise in practical measurements and inadequate for low-concentration gas inversion, the wavelength modulation approach is selected as the optimal measurement strategy.

To address the demands for environmental parameter monitoring in confined spaces, this study investigates high-precision simultaneous measurement of multi-component gases and multi-parameters. Based on TDLAS-WMS technology, absorption lines within four spectral bands (1381 nm for H_2_O, 2004 nm for CO_2_, 1570 nm for CO, and 760 nm for O_2_) were utilized to simultaneously detect environmental temperature, pressure, and concentrations of H_2_O, CO_2_, CO, and O_2_. A novel multi-parameter simultaneous inversion algorithm was developed, integrating Levenberg–Marquardt (L-M) fitting, single-line temperature and pressure measurement, spectral separation, and alternating iteration algorithms with an adaptive feedback mechanism. This approach achieved concurrent inversion of gas concentrations, temperature, and pressure, validated through numerical simulations. The simulation results show that the maximum error of temperature is 1.68%, the maximum error of pressure is 0.3% and the maximum errors of component concentrations of H_2_O, CO_2_, CO, O_2_ are 1.91%,2.49%,3.65% and 2.54%, respectively, with all errors remaining below 4%. These outcomes meet general requirements for measurement accuracy in practical applications.

## 2. Simultaneous Multi-Parameter Inversion Method

### 2.1. Basic Principle

According to Beer–Lambert’s law, when a light beam with frequency v and intensity $I_{0}$ passes through a gaseous medium, molecular photon absorption induces energy level transitions, resulting in attenuated transmitted intensity I(v) expressed as

(1) $$I(v)=I_{0}(v)\tau (v)=I_{0}(v)\exp[-PXS(T)\phi (v-v_{0})L]$$

where τ(v) denotes transmittance.

The absorbance α(v) is defined as

(2) $$\alpha (v)=\ln\frac{I_{0}(v)}{I(v)}=PXS(T)\phi (v-v_{0})L$$

Here, P (atm) is the total gas pressure. X is the volume fraction of target gas. L (cm) is the effective absorption pathlength. S(T) (cm^−2^ atm^−1^) is the absorption line strength at temperature T (K), and the line strength can be queried in the HITRAN database. $v_{0}$ (cm^−1^) is the center frequency of the absorption line. ϕ(v) is the normalized absorption line-shape function. This paper uses Voigt line-shape function. Usually, Voigt adopts the following [17]:

(3) $$\phi_{v}(v)=\frac{2}{\Delta v_{D}}\sqrt{\frac{\ln2}{\pi }}\frac{a}{\pi }\int_{-\infty }^{+\infty }\frac{\exp(-y^{2})}{a^{2}+{\left(w-y\right)}^{2}}dy$$

where $a=\sqrt{\ln2}\Delta v_{L}/\Delta v_{D}$, $w=2\sqrt{\ln2}(v-v_{0})/\Delta v_{D}$. $\Delta v_{D}$ is the full width at half maximum (FWHM) of a Gaussian line function, expressed as follows:

(4) $$\Delta v_{D}=2\sqrt{\ln2}\frac{v_{0}}{c}\sqrt{\frac{2kT}{M}}=7.1632\times 10^{-7}v_{0}\sqrt{\frac{T}{M}}$$

Here, c (cm/s) is the speed of light; M (g/mol) is the molar mass of gas molecules; *k* (J/K) is the Boltzmann constant. $\Delta v_{L}$ is the FWHM of the Lorentz line function, expressed as follows:

(5) $$\Delta v_{L}=P\sum_{j}X_{j}2\gamma_{j}(T)$$

where $X_{j}$ is the molar volume fraction of gas j; $\gamma_{j}(T)$ (cm^−1^/atm) is the collision broadening coefficient of gas j, $\gamma_{j}(T)=\gamma_{j}(T_{0})(T_{0}/T{)}^{n_{j}}$. $\gamma_{j}(T_{0})$ represents the collision broadening coefficient at temperature $T_{0}$, while $n_{j}$ is the temperature dependent coefficient that characterizes the relationship between collision broadening and temperature.

The integral absorbance A in the frequency domain is

(6) $$A=\int_{-\infty }^{+\infty }\alpha (\nu )d\nu =PXS(T)L$$

The gas concentration X is therefore derived as

(7) $$X=\frac{A}{PS(T)L}$$

Under high-frequency sinusoidal current modulation at frequency f, considering nonlinear responses of laser intensity and frequency to injection current, the temporal responses of DFB laser frequency and intensity with time *t* are

(8) $$v(t)=\bar{v}(t)+a\cos(2\pi ft+\varphi )$$

(9) $$I_{0}(t)={\bar{I}}_{0}[1+\sum_{m=1}^{\infty }i_{m}\cos(2m\pi ft+\varphi_{m})]$$

where $\bar{v}(t)$ (cm^−1^) is the center (mean) wavelength (or frequency) of the laser under modulation, a (cm^−1^) the modulation depth, φ the initial phase of the wavelength modulation, ${\bar{I}}_{0}$ the detector-measured laser intensity without modulation, $i_{m}$ is the $m^{th}$ Fourier coefficient of the measured detector signal with intensity modulation, and $\varphi_{m}$ is the initial phase of the $m^{th}$ order intensity modulation [18].

At the same time, $\tau \left(v\left(t\right)\right)$ can be expanded in the form of Fourier series with the modulation frequency f as the fundamental frequency, as follows:

(10) $$\tau \left(v\left(t\right)\right)=\sum_{k=0}^{\infty }\left[H_{k}\cos(k\cdot 2\pi ft)\right]$$

where $H_{k}$ is the $k^{th}$ order Fourier coefficient defined as

(11) $$H_{k}=\frac{1}{2\pi }\int_{-\pi }^{\pi }\tau [\bar{v}+a\cos\theta ]\cos k\theta d\theta$$

Using the modulated initial light intensity and transmittance, the expression of the transmitted light intensity can be obtained as follows:

(12) $$I_{t}(t)={\bar{I}}_{0}[1+\sum_{m=1}^{\infty }i_{m}\cos(2m\pi ft+\varphi_{m})]\cdot \sum_{k=0}^{\infty }\left[H_{k}\cos(k\cdot 2\pi ft)\right]$$

The transmitted light intensity signal $I_{t}(t)$ is multiplied by the cosine and sine reference signals with a frequency of nf, and then the high-frequency components are filtered out by the low-pass filter to retain the corresponding cosine DC component $X_{nf}$ and sine DC component $Y_{nf}$. The $X_{nf}$ and $Y_{nf}$ components of the $n^{th}$ harmonic signal are

(13) $$X_{nf}=\frac{{\bar{I}}_{0}}{2}\left[H_{n}+\frac{1}{2}\sum_{k=1}^{\infty }\left(H_{n+k}+\left(1+\delta_{nk}\right)H_{\left|n-k\right|}\right)i_{k}\cos\left(\varphi_{k}\right)\right]$$

(14) $$Y_{nf}=\frac{{\bar{I}}_{0}}{2}\left[\frac{1}{2}\sum_{k=1}^{\infty }\left(H_{n+k}-\left(1+\delta_{nk}\right)H_{\left|n-k\right|}\right)i_{k}\sin\left(\varphi_{k}\right)\right]$$

The raw harmonic signal WMS-*nf* is expressed as

(15) $$R_{nf}=\sqrt{X_{nf}^{2}+Y_{nf}^{2}}$$

When there is no absorption effect of gas on the light intensity, there is $H_{0}=1$, $H_{k}=0$. The $X_{nf}^{0}$ and $Y_{nf}^{0}$ components of the $n^{th}$ harmonic signal are called the background harmonic signal. The specific expression is as follows:

(16) $$X_{nf}^{0}=\frac{{\bar{I}}_{0}}{2}i_{n}\cos(\varphi_{n}).\;Y_{nf}^{0}=\frac{{\bar{I}}_{0}}{2}i_{n}\sin(\varphi_{n})$$

It can be seen that when there is no absorption in the optical path, there is also a non-zero background signal. The harmonic signal WMS-*nf* after subtracting the background can be expressed as

(17) $$S_{nf}=\sqrt{{\left(X_{nf}-X_{nf}^{0}\right)}^{2}+{\left(Y_{nf}-Y_{nf}^{0}\right)}^{2}}$$

The influence of light intensity jitter, non-absorption loss and other noise can be eliminated by using 1*f* signal to normalize *nf* signal. The normalized harmonic signal WMS-*nf*/l*f* after background subtraction can be expressed as

(18) $$S_{nf/1f}=\sqrt{{\left(\frac{X_{nf}}{R_{1f}}-\frac{X_{nf}^{0}}{R_{1f}^{0}}\right)}^{2}+{\left(\frac{Y_{nf}}{R_{1f}}-\frac{Y_{nf}^{0}}{R_{1f}^{0}}\right)}^{2}}$$

### 2.2. Harmonic Signal Fitting Algorithm

**Figure 1:**
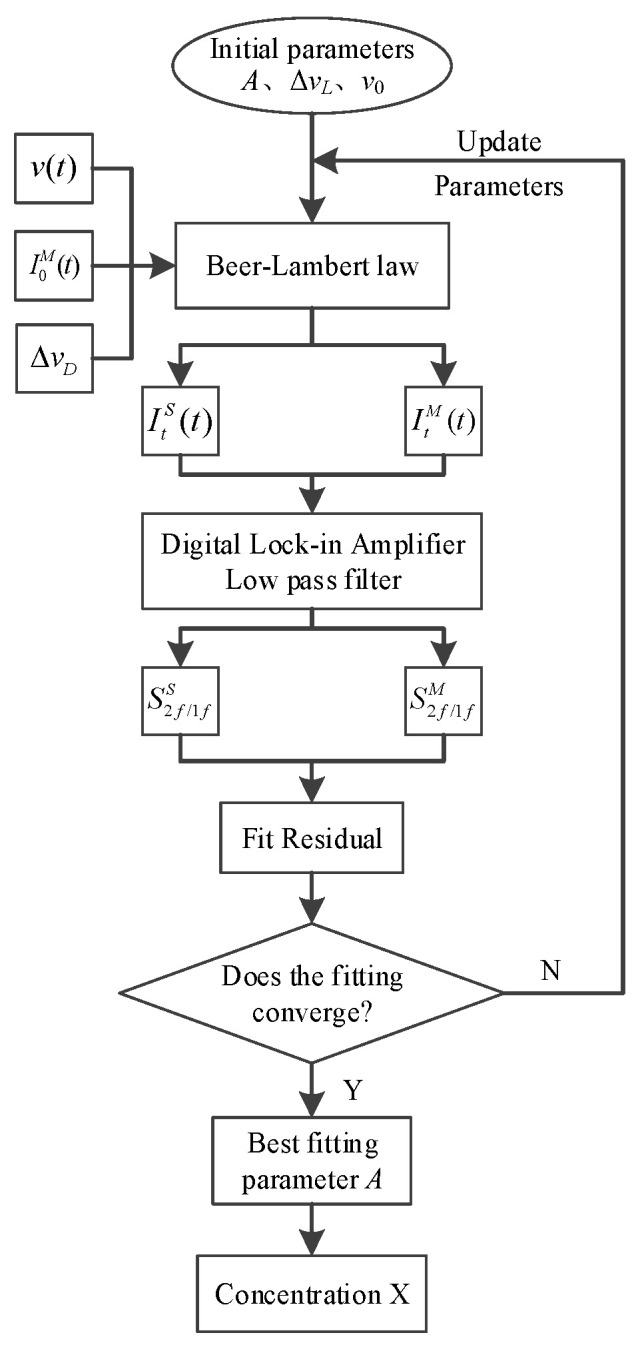
Workflow of calibration-free WMS algorithm via harmonic fitting.

Figure 1 illustrates the workflow of the calibration-free wavelength modulation algorithm based on harmonic fitting. The measured signal without gas absorption $I_{0}^{M}(t)$ is the background light intensity signal. Combined with the measured time-frequency response relationship v(t) and the set initial spectral parameter value, the spectral absorptivity curve is simulated and calculated. The simulated transmitted light intensity signal $I_{t}^{S}(t)$ is

(19) $$I_{t}^{S}(t)=I_{0}^{M}(t)\exp\{-A\varphi [v(t),v_{0},\Delta v_{L},\Delta v_{D}]\}$$

The simulated normalized harmonic signal $S_{2f/1f}^{S}$ can be extracted from the measured signal $I_{t}^{S}(t)$ according to the digital lock-in amplifier and the low pass filter. The least square method is used to fit $S_{2f/1f}^{M}$ with the integral absorption area A, the spectral line collision broadening $\Delta v_{L}$, the Doppler broadening $\Delta v_{D}$ (when the temperature is known, $\Delta v_{D}$ can be used as a constant without participating in the fitting process) and the center frequency *v*_0_ as the fitting parameters. When the residual between $S_{2f/1f}^{S}$ and $S_{2f/1f}^{M}$ can meet the set requirements, the fitting process converges. At this time, the best fitting parameter A can be obtained. Then, combined with the known quantities *T* and *P*, the gas concentration value can be calculated according to Equation (7).

### 2.3. Spectral Separation Algorithm

**Figure 2:**
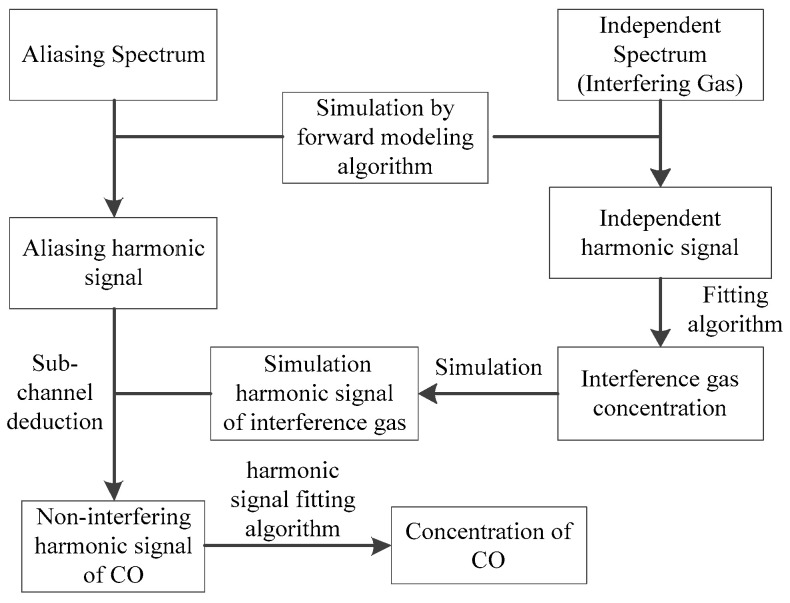
Workflow of spectral separation algorithm.

Aiming at the absorption spectrum with H_2_O, CO_2_, CO and O_2_ aliasing, the absorption information of H_2_O, CO_2_ and O_2_ is gradually separated by using the idea of reducing variables, and the effective absorption signal of CO is obtained. The specific process is shown in Figure 2. Firstly, the harmonic signal of the interfering gas is obtained by using the independent interfering gas absorption lines of other bands. According to the above fitting algorithm, the concentration of the interfering gas is obtained by inversion. Then, the harmonic signal of the interfering gas in the aliasing band is simulated by using the concentration of the interfering gas obtained by inversion. The influence of the interfering gas on the CO absorption is removed according to the sub-channel deduction method, and the separation of the aliasing spectrum is realized. The independent harmonic signal of CO is obtained, and the concentration of CO is obtained by fitting inversion.

### 2.4. Principle of Single-Line Thermometry and Manometry Algorithm

According to the principle, identical digital phase-locked and low-pass filtering are applied to both transmitted light intensity and the background signal, yielding X and Y components of the 2*f* harmonics of the absorption and background. The background-corrected second harmonic signal of the background is derived from Equation (17):

(20) $$S_{2f}=\sqrt{{\left(X_{2f}-X_{2f}^{0}\right)}^{2}+{\left(Y_{2f}-Y_{2f}^{0}\right)}^{2}}\approx \frac{{\bar{I}}_{0}}{2}[H_{2}-\frac{i_{1}}{2}(H_{1}+H_{3})]$$

By substituting $X_{2f}$, $Y_{2f}$, $X_{2f}^{0}$ and $Y_{2f}^{0}$ corresponding to Equation (13) into Equation (17) for calculation, Equation (20) can be simplified, where $H_{1}$, $H_{2}$, and $H_{3}$ are the Fourier expansion coefficients of 1st, 2nd, and 3rd orders corresponding to f Equation (11). For the isolated spectral line, at the absorption center, the odd term of the Fourier coefficient is 0, that is, $H_{1}$ = $H_{3}$ = 0. Therefore, the peak height of $S_{2f}$ can be simplified as

(21) $$p=S_{2f}(v_{0})\approx \frac{G{\bar{I}}_{0}}{2}H_{2}(v_{0})$$

The peak normalization of $S_{2f}$ is

(22) $$\begin{array}{cc} R_{2f/p} & =\frac{S_{2f}}{p}=\frac{H_{2}-\frac{i_{1}}{2}(H_{1}+H_{3})}{H_{2}(v_{0})}=\frac{\int_{-\pi }^{\pi }\phi_{j}(\Delta v_{L},\Delta v_{D},v(t)\cos(2\theta ))d\theta }{\int_{-\pi }^{\pi }\phi_{j}(\Delta v_{L},\Delta v_{D},v_{0}\cos(2\theta ))d\theta }\\ & -\frac{i_{1}}{2}\frac{\int_{-\pi }^{\pi }\phi_{j}(\Delta v_{L},\Delta v_{D},v(t)\cos(\theta ))d\theta +\int_{-\pi }^{\pi }\phi_{j}(\Delta v_{L},\Delta v_{D},v(t)\cos(3\theta ))d\theta }{\int_{-\pi }^{\pi }\phi_{j}(\Delta v_{L},\Delta v_{D},v_{0})\cos(2\theta ))d\theta } \end{array}$$

The peak-normalized 2*f* signal ($R_{2f/p}$) with the background removed eliminates the linear term $-PXLS(T)/(1+\delta_{0})\pi$ containing the concentration *X*, thereby eliminating the effect of concentration on $R_{2f/p}$. According to Equation (22), the signal $R_{2f/p}$ mainly depends on the collision broadening $\Delta v_{L}$ and Doppler broadening $\Delta v_{D}$ of the absorption lines. When the pressure remains constant, if suitable spectral lines are selected through simulation verification, so that the line shape of the spectral lines is not affected by concentration *X* and $R_{2f/p}$ is only related to temperature *T*, then it will be mainly affected by temperature *T*. Based on this characteristic, the temperature *T* of the gas can be solved using the signal $R_{2f/p}$. The specific simulation verification process can be found in Section 3.2. Due to the fact that this article is based on the TDLAS-WMS method, the changes in the absorption line shape here are mainly reflected in the variation of the sidelobe peak spacing of the harmonic signal.

**Figure 3:**
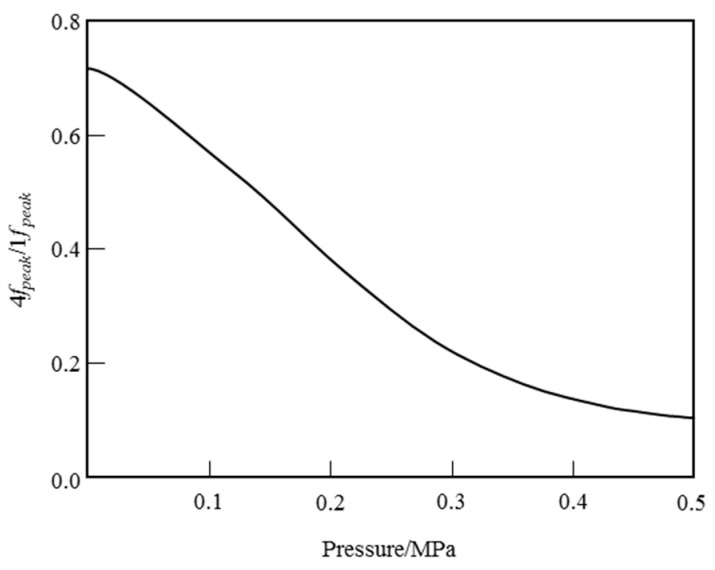
Variation of the peak ratio of the fourth harmonic signal and the second harmonic signal of a spectral line with pressure.

Through the simulation of harmonic signals using the constructed wavelength modulation spectroscopy model [19], it is evident that a monotonic functional relationship exists between the gas pressure and the ratio of the peak amplitude (*S*_4*f*, peak_/*S*_2*f*, peak_) of the fourth harmonic signal (*S*_4*f*_) to that of the second harmonic signal (*S*_2*f*_) of a single spectral line when the temperature concentration remains constant. This relationship can be described using a polynomial, as illustrated in Figure 3. Based on this monotonic relationship, a calibration-free measurement of pressure can be achieved. The specific simulation verification process can be found in Section 3.3.

### 2.5. Simultaneous Multi-Parameter Inversion Algorithm

**Figure 4:**
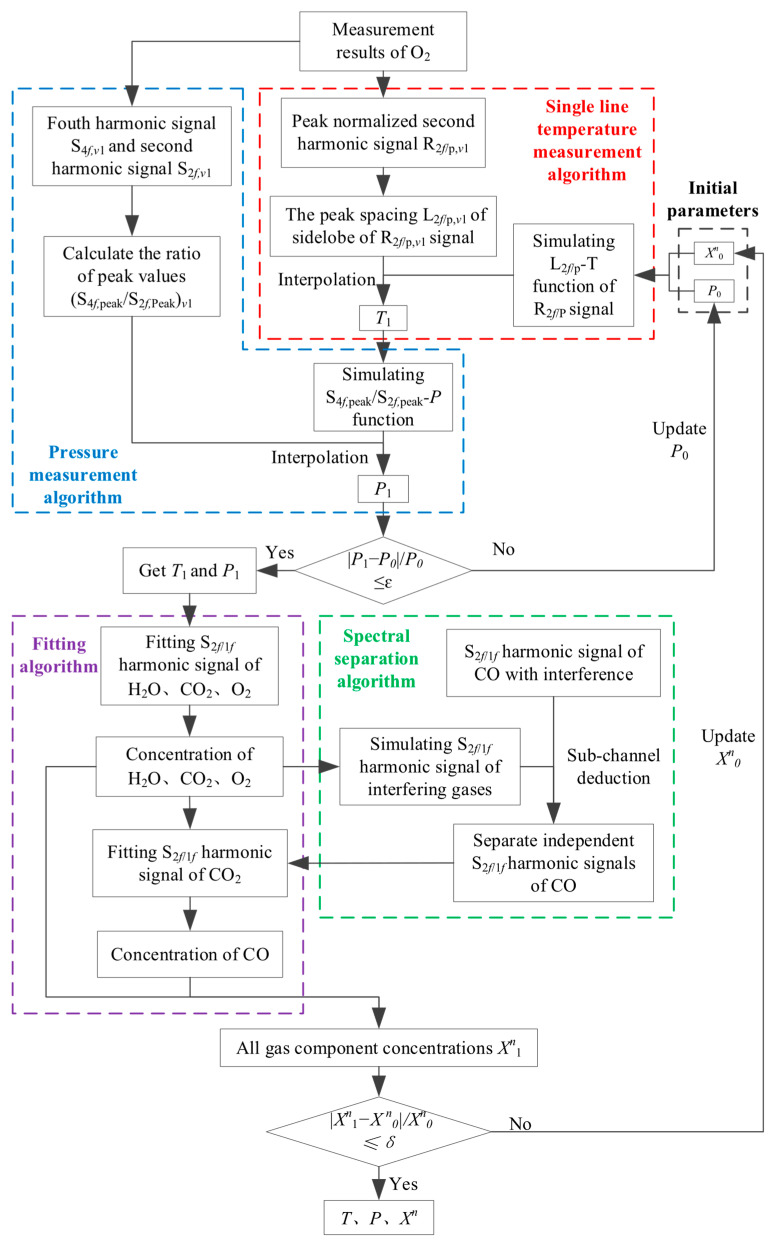
Workflow of simultaneous multi-parameter inversion algorithm.

Based on the calibration-free wavelength modulation method based on harmonic signal fitting (Section 2.2), in conjunction with the aforementioned calibration-free single-line thermometry and manometry methods (Section 2.4) and the composite absorption spectrum separation methods (Section 2.3), a multi-parameter simultaneous inversion algorithm is established. This algorithm can simultaneously invert parameters such as pressure, temperature, and gas component concentration. The temperature is determined using the single-line thermometry algorithm, which utilizes the background-subtracted peak-normalized 2*f* signal ($R_{2f/p}$). The pressure is determined using the single-line manometry algorithm, which relies on the ratio of the peak amplitude (*S*_4*f*, peak_/*S*_2*f*, peak_) of the fourth harmonic signal (*S*_4*f*_) to that of the second harmonic signal (*S*_2*f*_) for a given spectral line. Once temperature and pressure are obtained, a feedback condition between them is constructed. This condition constrains the iterative step direction to converge towards the local optimal values for temperature and pressure. Subsequently, based on these values, the *S*_2*f*/1*f*_ signals of all spectral lines are individually fitted to derive component concentrations. A constraint condition for O_2_ concentration relative to temperature and pressure is then established. The iterative steps are repeated, guided by these constraints, until convergence to the global optimal solution is achieved. This iterative process is illustrated in Figure 4.

The detailed steps are as follows:

(1) Extract the second harmonic signal *S*_2*f*_ and the fourth harmonic signal *S*_4*f*_ obtained by subtracting the background from the O_2_ molecule spectral line measured in the experiment. Calculate the peak normalized second harmonic signal $R_{2f/p}$ corresponding to the spectral line according to Equation (22), and calculate the corresponding sidelobe peak spacing.

(2) Predict the measured environment, given the initial pressure *P*_0_ and component concentration $X_{0}^{n}$.

(3) Set the temperature range, simulate the peak normalized second harmonic signal $R_{2f/p}$ of the O_2_ absorption line at different temperatures using a harmonic signal model based on the initial pressure *P*_0_ and initial oxygen concentration $X_{0}^{O_{2}}$, calculate the side lobe peak spacing of each harmonic signal, and establish a monotonic function relationship between sidelobe peak spacing and temperature.

(4) Compare the side lobe peak spacing of the $R_{2f/p}$ signal measured in the experiment in step (1) with the monotonic curve established in step (3), and interpolate to obtain the temperature *T*_1_.

(5) Based on the given initial parameters and temperature *T*_1_, set the pressure range, simulate the fourth harmonic signal *S*_4_*_f_* and second harmonic signal *S*_2_*_f_* corresponding to the O_2_ absorption line at different pressures using a harmonic signal model, calculate the ratio (*S*_4*f*,peak_/*S*_2*f*,peak_) of the two signal amplitudes, and establish a monotonic function relationship between *S*_4*f*,peak_/*S*_2*f*,peak_ and pressure.

(6) Using the second harmonic signal *S*_2_*_f_* and fourth harmonic signal *S*_4_*_f_* extracted in step (1), calculate the ratio (*S*_4*f*,peak_/*S*_2_*_f_*_,peak_) of the fourth harmonic signal amplitude (*S*_4*f*,peak_) to the second harmonic signal amplitude (*S*_2_*_f_*_,peak_) corresponding to the spectral line.

(7) Compare the experimentally measured values of *S*_4_*_f_*_,peak_/*S*_2_*_f_*_,peak_ obtained in step (6) with the monotonic curve established in step (5), and interpolate to obtain the pressure *P*_1_.

(8) If the condition $\left|P_{1}-P_{0}\right|/P_{0}\leq \varepsilon$ is satisfied, the iteration ends, and the temperature and pressure are obtained. If not, update *P*_0_, return to step (3), and re-iterate the calculation.

(9) According to the obtained temperature *T*_1_ and pressure *P*_1_, the WMS model is established with A, $\Delta v_{D}$ and $\Delta v_{L}$ as free variables. The *S*_2*f*/1*f*_ harmonic signals of H_2_O, CO_2_ and O_2_ are fitted by the least square method to obtain the parameters of A, $\Delta v_{D}$ and $\Delta v_{L}$ of the spectral lines, and the corresponding gas concentration is calculated according to Equation (7).

(10) The spectral separation algorithm is used to separate the composite absorption spectrum, and the independent CO harmonic signal is obtained and the CO concentration is obtained by inversion, and the component concentration $X_{1}^{n}$ of all gases is obtained.

(11) Determine whether the condition $\left|X_{1}^{n}-X_{0}^{n}\right|/X_{0}^{n}\leq \delta$ is met. If it is met, the iteration ends, and the temperature and pressure and gas component concentration are obtained. If it is not satisfied, update *P*_0_ and component concentration $X_{0}^{n}$, return to step (3), and re-iterate the calculation until the end of the iteration.

## 3. Algorithm Validation

### 3.1. Absorbing Line Selection

**Figure 5:**
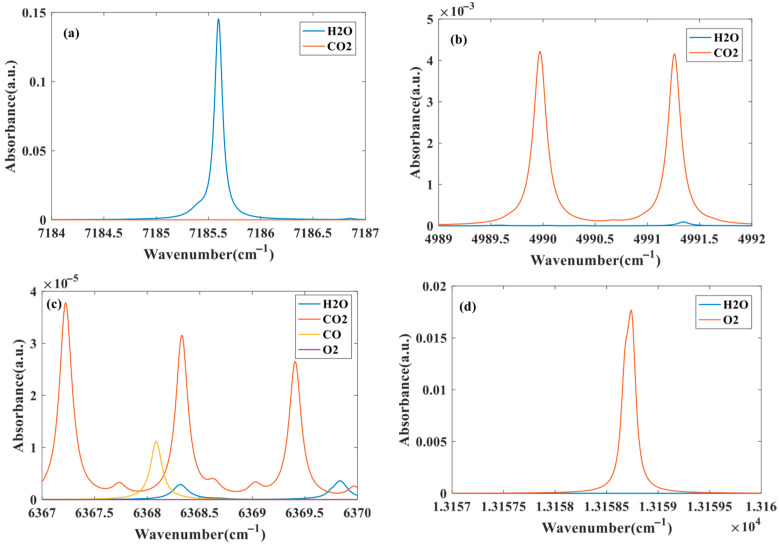
Simulation result of absorbance in (**a**) 1392 nm, (**b**) 2004 nm, (**c**) 1570 nm and (**d**) 760 nm band, when *T* = 298 K, *P* = 1 atm, *L* = 1 m.

In order to realize the simultaneous measurement of temperature, pressure and concentration of H_2_O, CO_2_, CO and O_2_, it is necessary to select the appropriate absorption line for scanning. Figure 5 shows the absorbance simulation results of H_2_O, CO_2_, CO and O_2_ gas molecules in the wave number range of 7184~7187 cm^−1^ (1392 nm laser), 4989~4992 cm^−1^ (2004 nm laser), 6367~6370 cm^−1^ (1570 nm laser) and 13,157~13,160 cm^−1^ (760 nm laser) at room temperature and atmospheric pressure.

It can be seen from Figure 5 that H_2_O, CO_2_ and O_2_ have obvious independent absorption in the ranges of 7184~7187 cm^−1^, 4989~4992 cm^−1^ and 13,157~13,160 cm^−1^, respectively, and other gas interference can be ignored, which can realize the inversion of gas component concentration and environmental parameters. Although there is gas interference in the range of 6367~6370 cm^−1^, the independent CO absorption can be separated by spectral separation algorithm, and then the CO concentration can be obtained by inversion. The specific spectral parameters obtained from HITRAN2020 are shown in Table 1, and the parameter values are directly obtained from HITRAN. Among them, the center frequency interval of the two absorption lines of H_2_O and O_2_ is small enough to be combined.

### 3.2. Effect of Temperature on $R_{2f/p}$ Signal

**Figure 6:**
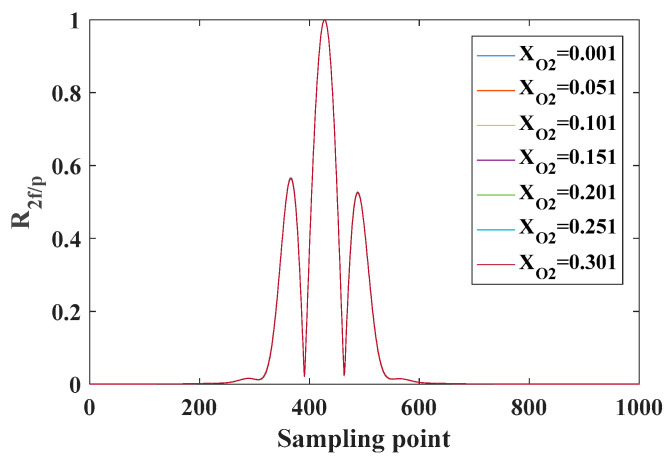
Simulation results of $R_{2f/p}$ at different O_2_ concentrations *X*_o2_ (the volume fraction of O_2_) in 760 nm band.

**Figure 7:**
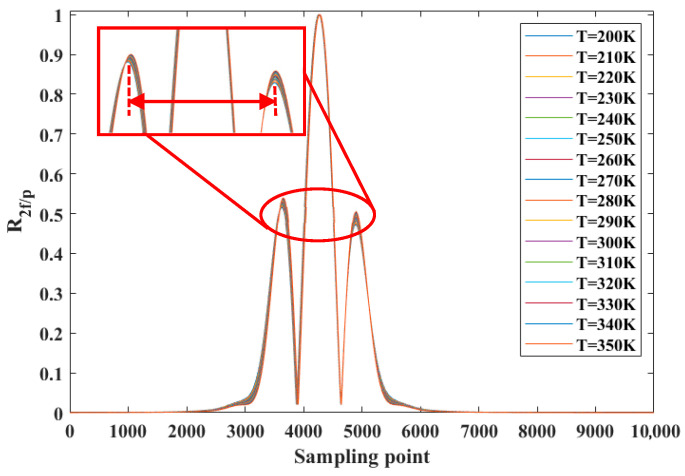
When the concentration of O_2_ remains unchanged, the simulation results of $R_{2f/p}$ at different temperatures in 760 nm band.

**Figure 8:**
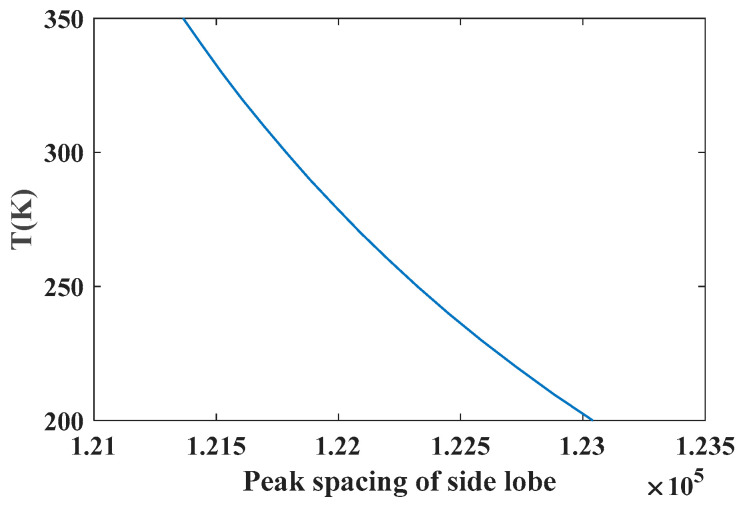
The variation of peak spacing between two sides of $R_{2f/p}$ with temperature.

According to the above single-line temperature measurement principle, the $R_{2f/p}$ signal of the absorption line required in the single-line method to solve the temperature is insensitive to concentration and sensitive to temperature. In this paper, the O_2_ absorption line in the 760 nm band is simulated and verified under the conditions of temperature of 25 °C, pressure of 1 atm and optical path of 1 m. A series of O_2_ concentration values are set, and the $R_{2f/p}$ signals at different concentrations are simulated by using the harmonic signal model, as shown in Figure 6. It can be seen from Figure 6 that the line-shape of $R_{2f/p}$ is not sensitive to concentration in a certain temperature range when the temperature and pressure are constant. When the O_2_ concentration remains unchanged, the line-shape of the $R_{2f/p}$ signal changes with temperature, as shown in Figure 7. It can be seen from Figure 7 that when the gas temperature changes within the range, the height and shape of the side lobes on both sides of the line-shape of the $R_{2f/p}$ signal change. It can be seen that the $R_{2f/p}$ line type corresponding to the O_2_ absorption line in the 760 nm band mainly depends on the temperature of the gas and is not sensitive to the change in the concentration. In addition, as shown by the enlarged curve in the red area of Figure 7, the peak spacing of the two side lobes of the absorption line corresponding to the $R_{2f/p}$ line type (indicated by the red arrow) changes with the change in temperature. Setting the temperature range to 200 K~350 K, the peak spacing of each side lobe corresponding to each temperature point is obtained, and the curve of the peak spacing of the side lobe with temperature is shown in Figure 8. It can be seen that there is a monotonic relationship between the peak spacing of side lobes and temperature. Temperature measurement can be achieved by using the peak spacing of side lobes corresponding to the $R_{2f/p}$ line type of the O_2_ absorption line in the 760 nm band.

### 3.3. Effect of Pressure on $S_{4f,peak}/S_{2f,peak}$

**Figure 9:**
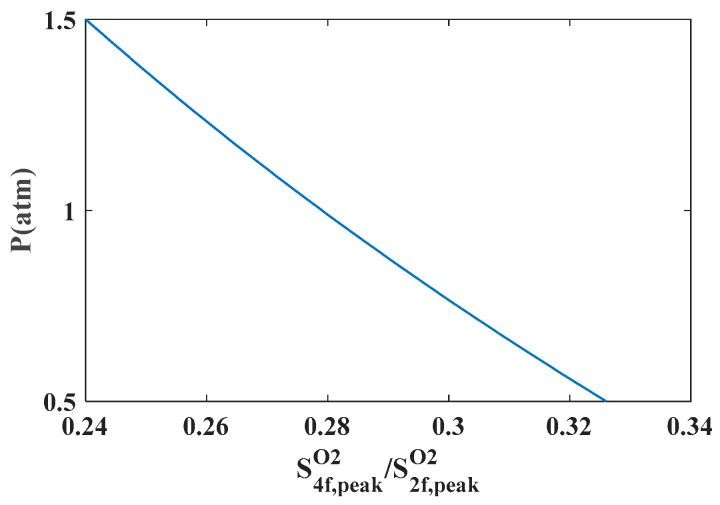
Variation of the peak ratio of the fourth harmonic signal and the second harmonic signal of 760 nm band with pressure.

According to the above pressure measurement method, there is a monotonic function relationship between the gas pressure and the ratio of the fourth harmonic signal (*S*_4*f*_) amplitude of the absorption line to the second harmonic signal (*S*_2*f*_) amplitude (*S*_4*f*,peak_/*S*_2*f*,peak_) in the process of solving the pressure. In this paper, the O_2_ absorption line in the 760 nm band is simulated and verified under the conditions of temperature of 25 °C and optical path of 1 m. The pressure range is set to 0.5 atm~1.5 atm, and the harmonic signal model is used to simulate the fourth harmonic signal *S*_4*f*_ and the second harmonic signal S_2*f*_ corresponding to the O_2_ absorption line in the 760 nm band under different pressures. The ratio of the amplitude (*S*_4*f*,peak_/*S*_2*f*,peak_) is calculated, and the monotonic function relationship between *S*_4*f*,peak_/*S*_2*f*,peak_ and pressure is established, as shown in Figure 9. It can be seen that the O_2_ absorption line in the 760 nm band meets the conditions of pressure measurement and can achieve pressure measurement.

### 3.4. Validation of Simultaneous Multi-Parameter Inversion Algorithm

**Figure 10:**
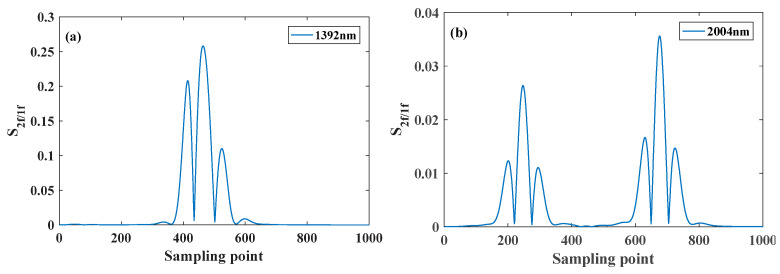

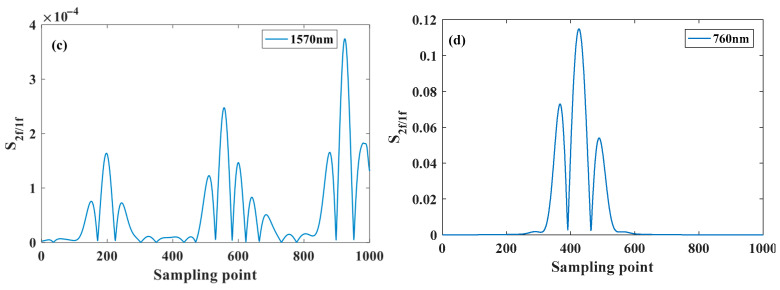
Simulation measurement data for (**a**) H_2_O, (**b**) CO_2_, (**c**) CO and (**d**) O_2_ in 1381 nm, 2004 nm, 1570 nm and 760 nm bands.

**Figure 11:**
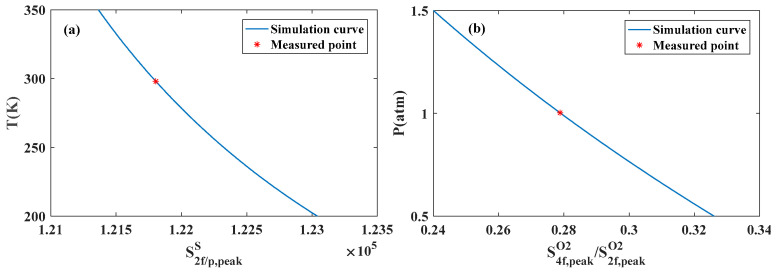
Simulation results of measurement of (**a**) temperature and (**b**) pressure in 760 nm bands.

**Figure 12:**
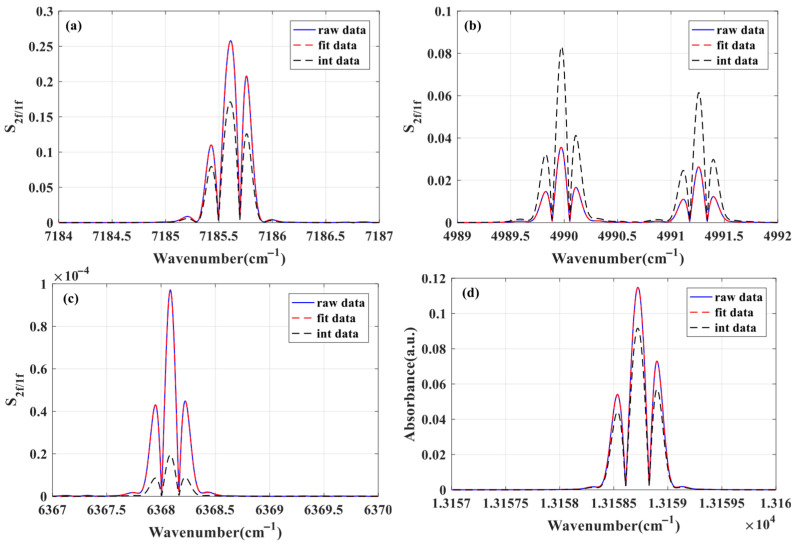
Simulation results of harmonic signal fitting of (**a**) H_2_O, (**b**) CO_2_, (**c**) CO and (**d**) O_2_.

According to the above multi-parameter simultaneous inversion algorithm, under the condition of temperature of 25 °C, pressure of 1 atm and optical path of 1 m, the simultaneous inversion of temperature, pressure and concentration of H_2_O, CO_2_, CO and O_2_ components is simulated and verified by using the corresponding absorption lines in the four bands of 1381 nm, 2004 nm, 1570 nm and 760 nm. Firstly, the spectral model is used to simulate the actual measurement process, and the simulated measurement data are obtained, as shown in Figure 10. Then, according to the above algorithm steps, the monotonic function relationship between the side lobe peak spacing of the 760 nm O_2_ absorption line and the temperature is established by using the initial value parameters, and the temperature *T*_1_ is obtained by interpolation, as shown in Figure 11a. According to the given initial parameters and temperature *T*_1_, the monotonic function relationship between *S*_4*f*,peak_/*S*_2*f*,peak_ and pressure is established, and the pressure *P*_1_ is obtained by interpolation, as shown in Figure 11b. Repeat the above steps to iterate until the pressure meets the iterative conditions, and the temperature and pressure results at this time are obtained. The *S*_2*f*/1*f*_ harmonic signals of H_2_O, CO_2_ and O_2_ measured are fitted by the least square method to obtain the corresponding component concentration. The spectral separation algorithm is used to separate the composite absorption spectrum of the 1570 nm band to obtain an independent CO harmonic signal and invert the CO concentration, as shown in Figure 12. Repeat the above process, update the inversion parameters until the iterative conditions are met, and finally the temperature, pressure, and component concentration results are obtained.

### 3.5. Simulation Results and Analysis

In order to verify the reliability of the multi-parameter simultaneous inversion algorithm, we select different temperature, pressure and component concentration values within their measurement range, set different initial fitting values, and analyze their errors obtained by simultaneous inversion. In order to ensure the accurate measurement of H_2_O, CO_2_, CO and O_2_ in the ground conventional environment, the temperature measurement range is set from −40 °C to 40 °C, and the corresponding maximum H_2_O concentration measurement range contained in the air is from 1 × 10^−4^ to 5 × 10^−2^. The pressure in the conventional environment is generally maintained at about 1 atm, and the pressure measurement range is set from 0.8 atm to 1.2 atm. Since the concentration of CO_2_ in the air accounts for about 0.03%, in order to achieve the coverage of this concentration, the measurement range of CO_2_ concentration is set from 5 × 10^−5^ to 5.5 × 10^−4^. The content of O_2_ in the air accounts for about 21%. In order to achieve the coverage of this concentration, the measurement range of O_2_ concentration is set from 0.1 to 0.3. In general, the maximum concentration of CO that adults can withstand is about 5 × 10^−5^, and the measurement range of CO concentration is set from 5 × 10^−5^ to 5.5 × 10^−4^. The optical path length is set to 1 m. In the measurement range, five groups of temperature, pressure and concentration values are taken as the true values for simulation, and the values within one tenth of each set of true values are set as initial values input into the algorithm to invert the temperature, pressure and gas component concentration, so as to realize the multi-parameter simultaneous measurement of the ground conventional environment.

**Figure 13:**
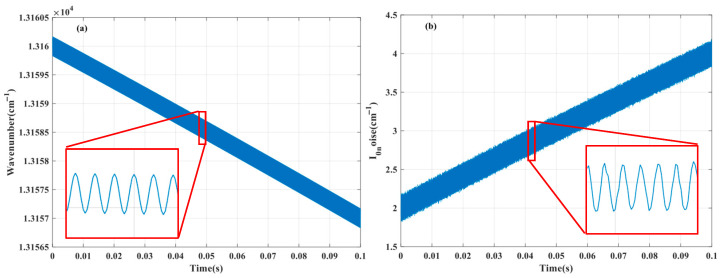
After adding interference, (**a**) the simulated modulation frequency response of the laser output and (**b**) the simulated light intensity signal of the laser output.

In addition, in order to make the simulated data (as shown in Figure 10) closer to the actual measured data, we analyzed the interference present in the TDLAS-WMS system. In the actual measurement process, the interference of the experimental system mainly includes noise interference and non-ideal characteristics of the system. The system noise mainly includes detector thermal noise, 1/f noise from laser drivers and lock-in amplifiers, and random phase disturbances caused by mechanical vibrations. The non ideal characteristics of the system are mainly affected by residual amplitude modulation (RAM) and nonlinear response of laser output frequency. Taking into full consideration the aforementioned interference factors, we separately added the aforementioned interference to the ideal simulation data (as shown in Figure 10). Firstly, we added a nonlinear term to the unmodulated frequency scanning signal generated during the simulation process, reflecting the nonlinear response of the laser output frequency. Then, we added noise to the modulation amplitude of the modulated frequency scanning signal to simulate the residual amplitude modulation (RAM) situation, as shown in Figure 13a. Next, random phase noise was added to the modulated light intensity signal to simulate mechanical vibrations during actual measurements; Finally, considering the influence of thermal noise and shot noise in the experimental environment, Gaussian white noise (signal-to-noise ratio of 50 dB) was added to the modulated light intensity signal, as shown in Figure 13b. When considering actual measurements, a series of noise reduction processes will be applied to the measurement signal, so adding Gaussian white noise results in a relatively high signal-to-noise ratio. In addition, WMS technology theoretically suppresses 1/f noise, so its impact is ignored. Then, the multi-parameter inversion algorithm was validated using simulation data with added interference noise as input. We validated the five experimental groups mentioned above in sequence. Each experimental group randomly generated initial values within one tenth of the true value range and we conducted five repeated tests. The average results and corresponding errors of each experimental group were recorded in Table 2.

**Figure 14:**
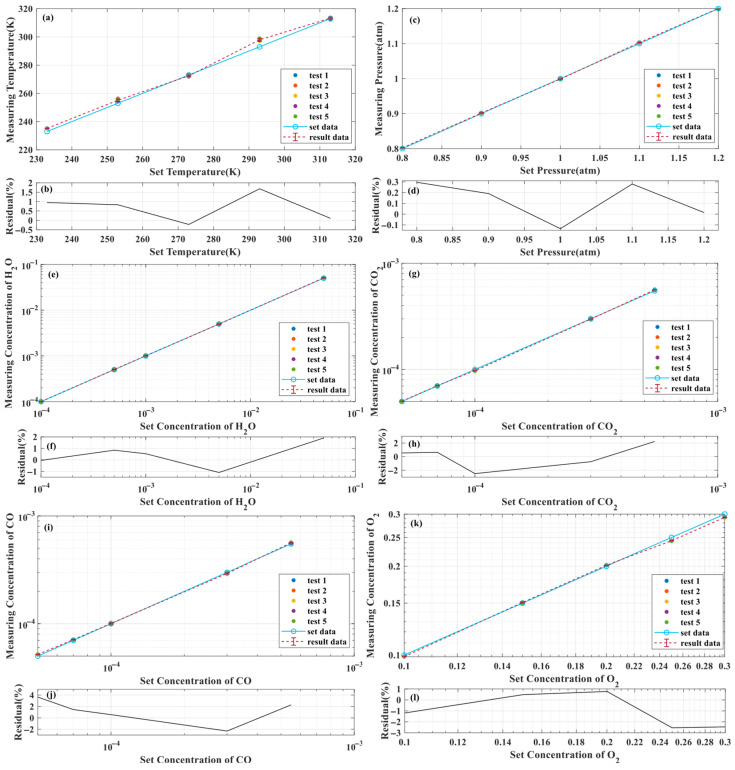
The five repeated test results and corresponding error bars (standard deviations) for (**a**) temperature, (**c**) pressure and component concentrations of (**e**) H_2_O, (**g**) CO_2_, (**i**) CO and (**k**) O_2_; and the error between the average inversion results and the true values of (**b**) temperature, (**d**) pressure and component concentrations of (**f**) H_2_O, (**h**) CO_2_, (**j**) CO and (**l**) O_2_.

Based on the results of five tests (with different initial values) conducted in each of the five experimental groups, the average results and corresponding standard deviations of each experimental group were calculated, as shown in the “result data” curves in Figure 14a,c,e,g,i,k. Points test1 to test5 are the results of the five tests, and the “set data” curves are the set true values. Figure 14b,d,f,h,j,l show the residuals between the average results and the true values of the five experimental groups. According to the statistical results in Table 2 and Figure 14, the error between the average inversion result of temperature and the true value is less than 2.0%, with a maximum value of 1.68%. The error between the average inversion result of pressure and the true value is less than 0.3%. The error between the average inversion result for H_2_O concentration and the true value is less than 2.0%, with a maximum value of 1.91%. The error between the average inversion result of CO_2_ concentration and the true value is less than 2.5%, with a maximum value of 2.49%. The error between the average inversion result for CO concentration and the true value is less than 4.0%, with a maximum value of 3.65%. The error between the average inversion result of O_2_ concentration and the true value is less than 3.0%, with a maximum value of 2.54%. Overall, the inversion error of CO concentration is the largest among several inversion parameters, which is due to the error propagation and accumulation in the algorithm’s principle. From Figure 5c, it can be seen that there is interference in the CO absorption spectrum, and spectral separation processing is needed to separate the pure harmonic signal of CO. The spectral separation algorithm constructed in this article requires the use of concentration inversion results of other interfering gases, causing their inversion errors to ultimately converge on the CO concentration. This is reflected in the data as a relatively high inversion error for CO. However, even with noise and interference added to the input signal to simulate a realistic experimental environment, the overall inversion error of the algorithm in this paper remains within 4.0%, meeting the measurement accuracy requirements for general applications. Therefore, the proposed method exhibits reliable performance throughout the entire measurement range, capable of simultaneously inverting temperature, pressure, and gas concentration, providing an effective method for multi-parameter measurements under normal ground environmental conditions. In the future, we will further analyze the sources and propagation principles of algorithm errors, and consider introducing relevant algorithms such as metaheuristics to further improve the overall performance of the algorithm and reduce the propagation and generation of errors.

## 4. Conclusions

In this paper, a multi-parameter simultaneous inversion method based on TDLAS-WMS technology is proposed. By innovatively integrating the L-M fitting algorithm, single-line thermometry and manometry methods, spectral separation, and alternating iteration algorithms with an adaptive feedback mechanism, simultaneous inversion of gas concentrations, temperature, and pressure is achieved and validated through simulations. The simulation results show that the maximum error of temperature is 1.68%, the maximum error of pressure is 0.3% and the maximum errors of component concentrations of H_2_O, CO_2_, CO, O_2_ are 1.91%,2.49%,3.65% and 2.54%, respectively. All errors remain within 4%, meeting the measurement accuracy requirements for general applications. This provides an effective monitoring solution for multi-parameter measurements in confined spaces under normal environmental conditions and lays the foundation for broader applications of TDLAS technology in personnel safety assurance and confined space monitoring.

## Figures and Tables

**Table 1:** Spectral line parameters.

Gas	Row Number	$v_{0}$ ^1^ (cm^−1^)	$S_{0}$ ^2^ (cm^−2^atm^−1^)	$\gamma_{air}$ ^3^ (cm^−1^atm^−1^)	$\gamma_{self}$ ^4^ (cm^−2^atm^−1^)	$E^{\prime \prime }$ ^5^ (cm^−1^)	$n_{T}$ ^6^	$P_{shift}$ ^7^ (cm^−1^)
H_2_O	74,282	7185.596113	0.014802	0.0413	0.195	1045.058	0.65	−0.02198
	74,283	7185.596433	0.004934	0.0388	0.371	1045.058	0.41	−0.02202
CO_2_	120,226	4989.971361	0.032728	0.0740	0.100	106.1297	0.70	−0.00448
CO	995	6368.086139	0.000464	0.0651	0.071	38.4481	0.74	−0.0051
O_2_	1826	13,158.678972	5.08 × 10^−5^	0.0408	0.042	546.7042	0.74	−0.00845
	1827	13,158.743572	8.31 × 10^−5^	0.0423	0.044	438.4413	0.68	−0.00877

^1^ Line position, parameter uncertainty (cm^−1^): (1) H_2_O: >10^−7^ and <10^−6^; (2) CO_2_: >10^−3^ and <10^−2^; (3) CO: >10^−5^ and <10^−4^; (4) O_2_: >10^−6^ and <10^−5^. ^2^ Spectral line intensity (T = 296 K), parameter uncertainty: (1) H_2_O: >2% and <5%; (2) CO_2_: >1% and <2%; (3) CO: >1% and <2%; (4) O_2_: <1%. ^3^ Air-broadening half-width for Lorentz profile, parameter uncertainty: (1) H_2_O: >10% and <20%; (2) CO_2_: >2% and <5%; (3) CO: >5% and <10%; (4) O_2_: >1% and <2%. ^4^ Self-broadening half-width for Lorentz profile, parameter uncertainty: (1) H_2_O: average or estimate; (2) CO_2_: >2% and <5%; (3) CO: >2% and <5%; (4) O_2_: >1% and <2%. ^5^ Lower-state energy of the transition. ^6^ Temperature dependence exponent, parameter uncertainty: (1) H_2_O: average or estimate; (2) CO_2_: >2% and <5%; (3) CO: >2% and <5%; (4) O_2_: >10% and <20%. ^7^ Pressure shift of the line position, parameter uncertainty (cm^−1^): (1) H_2_O: >0.1 and <1; (2) CO_2_: >10^−3^ and <10^−2^; (3) CO: >10^−3^ and <10^−2^; (4) O_2_: >10^−5^ and <10^−4^.

**Table 2:** The average result and error of multi-parameter simultaneous inversion algorithm.

Test Group	Parameter	Temperature (K)	Pressure (atm)	Concentration of H_2_O	Concentration of CO_2_	Concentration of CO	Concentration of O_2_
1	True value	233	0.8	1 × 10^−4^	5 × 10^−5^	5 × 10^−5^	0.1
	Average result	235.216	0.8024	0.999 × 10^−4^	5.027 × 10^−5^	5.183 × 10^−5^	0.098
	Deviation(%)	0.95	0.30	−0.04	0.54	3.65	−1.19
2	True value	253	0.9	5 × 10^−4^	7 × 10^−5^	7 × 10^−5^	0.15
	Average result	255.103	0.9017	5.042 × 10^−4^	7.045 × 10^−5^	7.102 × 10^−5^	0.151
	Deviation(%)	0.83	0.19	0.84	0.64	1.46	0.48
3	True value	273	1.0	1 × 10^−3^	1 × 10^−4^	1 × 10^−4^	0.20
	Average result	272.411	0.9987	1.005 × 10^−3^	9.751 × 10^−4^	1.006 × 10^−4^	0.202
	Deviation(%)	−0.22	−0.14	0.54	−2.49	0.57	0.77
4	True value	293	1.1	5 × 10^−3^	3 × 10^−4^	3 × 10^−4^	0.25
	Average result	297.934	1.1031	4.945 × 10^−3^	2.978 × 10^−4^	2.931 × 10^−4^	0.244
	Deviation(%)	1.68	0.28	−1.09	−0.74	−2.30	−2.54
5	True value	313	1.2	5 × 10^−2^	5.5 × 10^−4^	5.5 × 10^−4^	0.30
	Average result	313.338	1.2002	5.095 × 10^−2^	5.623 × 10^−4^	5.625 × 10^−4^	0.293
	Deviation(%)	0.11	0.02	1.91	2.24	2.28	−2.46

## Data Availability

The spectral data presented in the study are openly available in the HITRAN dataset at https://hitran.org/ (accessed on 17 June 2024).
